# Imaging features of biliary adenofibroma of the liver with malignant transformation: a case report with literature review

**DOI:** 10.1186/s12880-022-00775-9

**Published:** 2022-03-17

**Authors:** Wenjun Hu, Ying Zhao, Yunsong Liu, Zhengyu Hua, Ailian Liu

**Affiliations:** 1 Department of Radiology, The First Affiliated Hospital, Dalian Medical University, Dalian, Liaoning China; 2 Department of Pathology, The First Affiliated Hospital, Dalian Medical University, Dalian, Liaoning China; 3 Dalian Engineering Research Center for Artificial Intelligence in Medical Imaging, Dalian, Liaoning China

**Keywords:** Biliary adenofibroma, Malignant transformation, Imaging features

## Abstract

**Background:**

Biliary adenofibroma (BAF) is a rare primary hepatic tumor with the potential risk of malignant transformation. Given the extreme rarity of the disease, the imaging features of BAF are unclear. We presented a case of malignant BAF and conducted a systematic literature review. We highlighted the key imaging features in the diagnosis and aggressiveness assessment of BAF, as well as the role of various imaging modalities in evaluating BAF.

**Case presentation:**

We reported a 64-year-old woman with a 5-months history of pain in the right upper quadrant abdomen. US of the liver showed a hypoechoic subcapsular nodule. CT scan revealed a subcapsular solid-cystic mass in segment V of the liver. The mass showed a marked enhancement in the arterial phase followed by wash-out in the venous phase. The patient underwent partial resection of liver’s right lobe. The mass was diagnosed as BAF with malignant transformation by postoperative pathology.

**Conclusions:**

CT and MRI are helpful in recognizing and characterizing BAF. The imaging features of BAF include a solitary, large solid-cystic mass with a well-defined margin, lobulated shape, and internal septa; subcapsular location; no intrahepatic bile duct communication; the presence of von Meyenberg complexes in background liver. The enhancement patterns may have the potential to assess the aggressiveness of BAF, and that marked enhancement in the arterial phase followed by wash-out in the venous phase is suggestive of malignant BAF.

## Background

Biliary adenofibroma (BAF) is a rare primary hepatic tumor characterized by tubulocystic glandular structures and abundant fibroblastic stroma [1]. Although usually benign, it tends to recur after subtotal excision and has a potential for malignant transformation. To date, only 12 cases of malignant BAF have been reported in the medical literature. Accurate preoperative imaging diagnosis of premalignant lesions is essential for selecting appropriate treatments. However, very few reports focused on imaging features of BAF. Here, we reported a BAF case with malignant transformation and conducted a systematic review of the literature published between 1993 and 2019. We emphasized the key imaging features in the diagnosis and aggressiveness assessment of BAF, as well as the role of various imaging modalities in evaluating BAF.

## Case presentation

A 64-year-old woman was admitted to our hospital with a 5-months history of dull pain in the right upper quadrant abdomen. The pain was intermittent without obvious incentive. She had no fever, nausea, vomiting, or hematemesis. The physical examination was unremarkable. Alcohol and nicotine consumption was denied. The family history was non-contributory. Notably, she had a history of hepatitis B virus infection. Hepatitis B surface antigen, e antibody, core antibody were positive. Complete blood count, chemistry, coagulation, and liver function tests were within the normal limits. The tumor markers including AFP、CA125、CA19-9、CA15-3 and CA72-4 were not elevated.

**Fig. 1:**
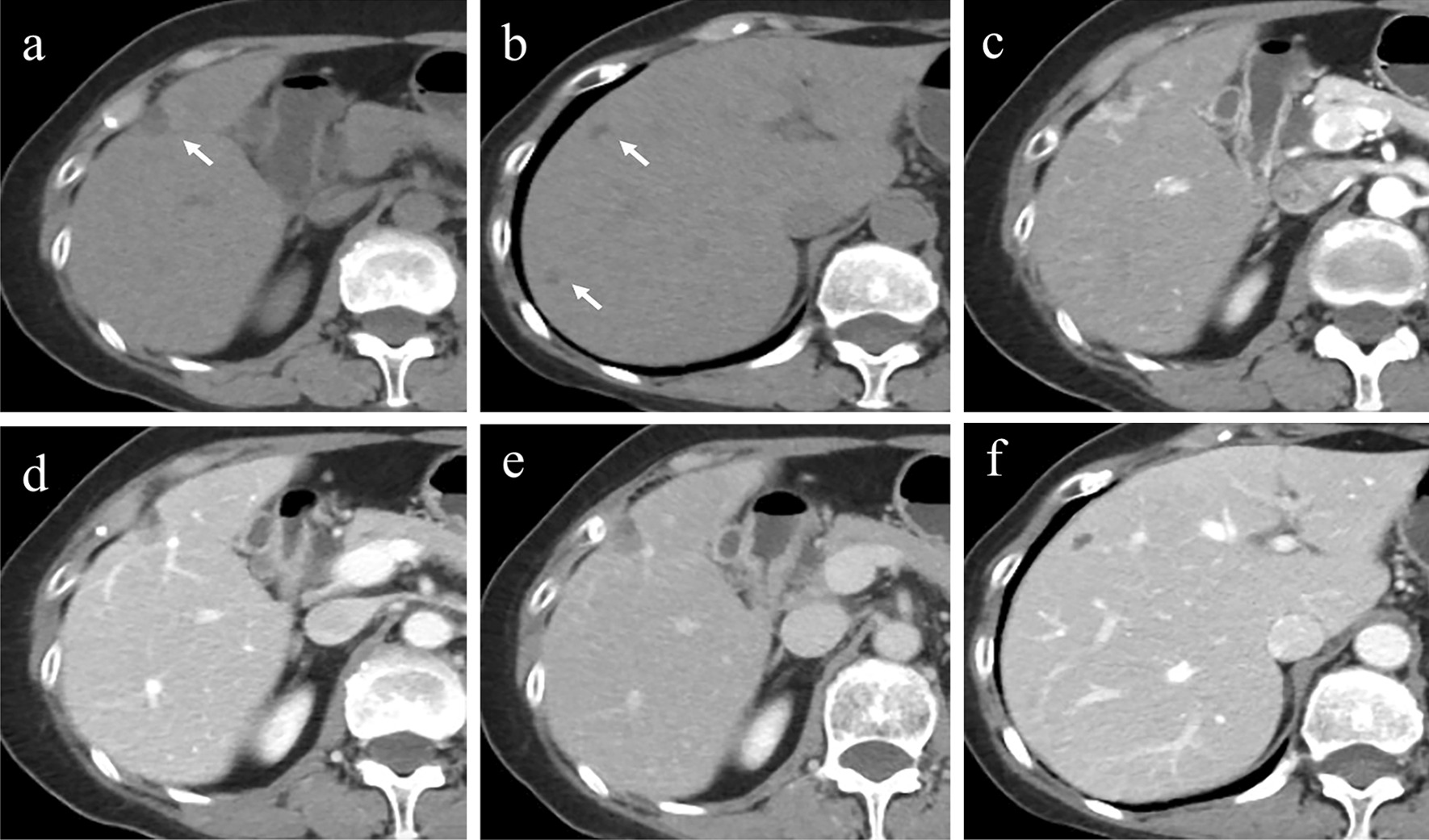
**a** Abdominal plain CT scan revealed a hypodense subcapsular solid-cystic mass (white arrow) with a well-defined boundary in liver segment IV. The adjacent hepatic capsule retraction can be seen. **b** Multiple small cystic lesions (white arrows) with irregular margins were scattered in the subcapsular area. The mass showed markedly heterogeneous enhancement in the **c** arterial phase followed by washout in the **d** venous and **e** delayed phases. **f** No enhancement was observed in those small cystic lesions during the venous phase

MRI scan obtained at other hospital revealed a 1.6 × 1.2 cm mass in the right liver lobe. Ultrasound (US) of the liver showed a hypoechoic subcapsular nodule of 20 × 11 mm with a well-defined margin, regular shape, and light vascularity. An abdominal plain CT scan revealed a hypodense mass measuring 1.8 × 1.3 cm underneath the liver capsule of the segment V, and the average CT value was 34 HU. An area along the left border of the mass showed even lower density, with CT values averaging 18 HU. The mass had a well-defined boundary and caused retraction of the adjacent hepatic capsule (Fig. 1a). In addition, multiple small cystic lesions with irregular margins were scattered in both lobes of the liver, especially in the subcapsular area. The largest one was 0.5 cm in diameter (Fig. 1b). After intravenous contrast medium injection, the mass showed a marked enhancement in the arterial phase followed by wash-out in the venous phase. The lower density area showed slight enhancement (Fig. 1c–e). No enhancement was seen in those small cystic lesions (Fig. 1f). Intrahepatic bile ducts looked normal. There were no enlarged lymph nodes.

**Fig. 2:**
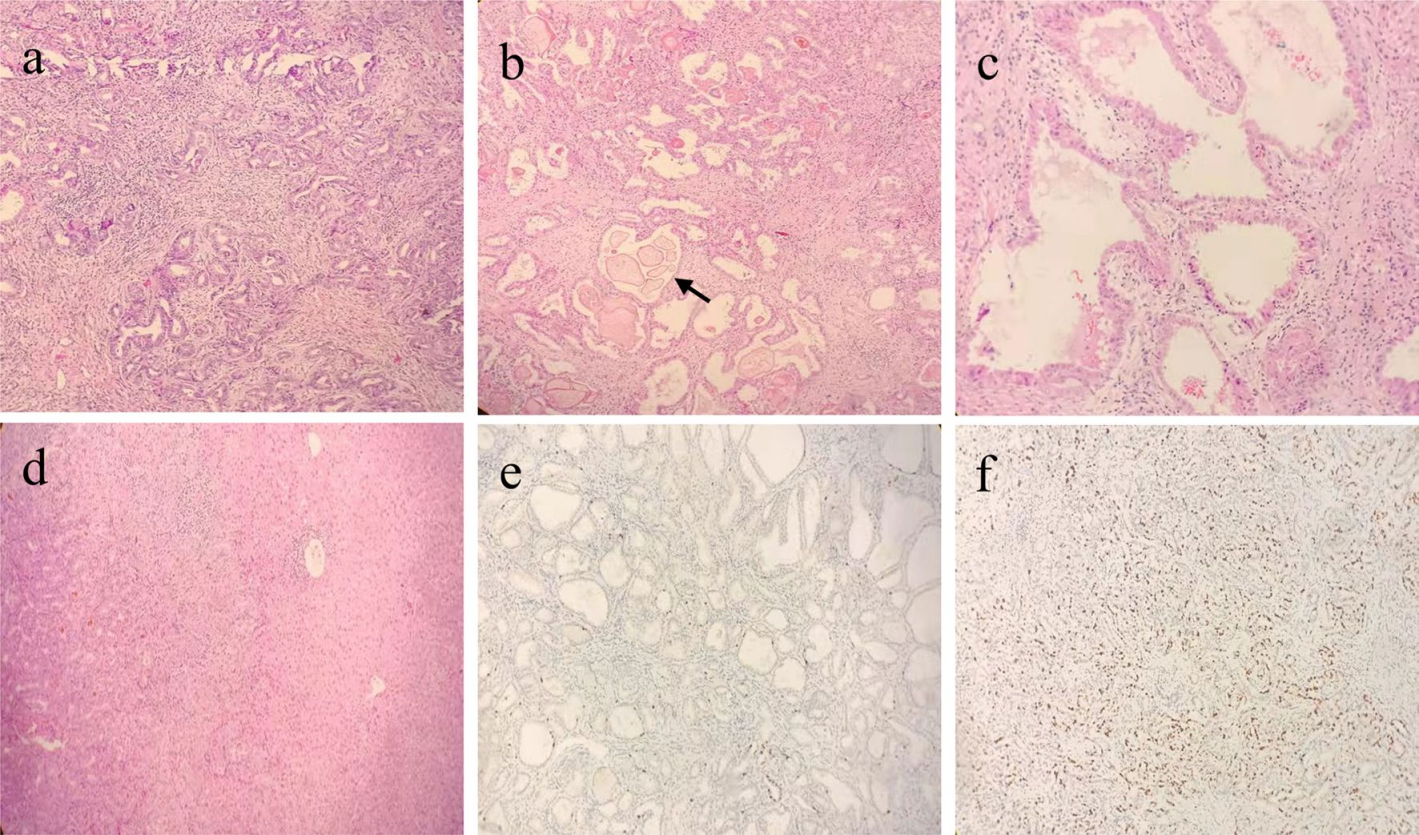
**a** Cuboidal to short columnar tumor cells were arranged in tubuloglandular structures, and some tubules dilated to cysts. These tubulocystic structures set in a fibrous stroma (H&E × 100). **b** Bile-like materials were observed in some lumens of the tubulocystic structures (black arrow)(H&E × 100). **c** Apocrine-like changes were seen in some lining epithelial cells. **d** Irregular tubules were densely arranged, the tumor cells showed round nuclei with prominent nucleoli and distinct nuclear membrane (left side; adenocarcinoma). And invasive growth in the adjacent liver parenchyma (right side) could be seen (H&E × 100). **e** Ki67 proliferation index in the benign part of the tumor was less than 10% (Ki67 staining × 100). **f** Ki67 proliferation index in the malignant part of the tumor markedly increased to 20–30% (Ki67 staining × 100)

The patient subsequently underwent partial resection of liver’s right lobe. Her postoperative course was uneventful and she was discharged on postoperative day 5. Gross examination of the resection specimen revealed a 1.5 × 1.5 × 1.2 cm subcapsular firm mass with irregular outline and whitish surface. Histological examination showed that the lesion was composed of tubular structures embedded in a fibrous stroma and the tubules had variable sizes and irregular shapes, with some of them dilated to cysts (Fig. 2a). Bile-like materials were observed in some lumens of the tubulocystic structures (Fig. 2b). The epithelial lining was a single layer of cuboidal to low columnar cells and apocrine-like changes were seen in some areas (Fig. 2c). A part of the lesion showed crowded tubular structures with closely packed nuclei. In these areas, the nucleoli were prominent and the nuclear membrane showed distinct contour. Mitotic figures could be easily detected. And invasive growth in the adjacent liver parenchyma could be seen focally (Fig. 2d). Immunohistochemically, the epithelial cells stained positive for CK7、CK19、CEA. Ki67 proliferation index in the benign part of the tumor was less than 10% (Fig. 2e), and that of the malignant part was 20–30% (Fig. 2f). Based on the histopathological result, the final diagnosis was BAF with malignant transformation (middle to well-differentiated adenocarcinoma).

## Discussion and conclusion

In 1993, BAF was first described by Tsui et al. [1] as a tubulocystic hepatic tumor with abundant fibrous stroma. The WHO classification classified BAF as a benign tumor originating from bile duct [2]. However, of the 25 prior cases of BAF reported in the literature, 12 were associated with evidence of malignant transformation. In addition, abnormalities of chromosome 22 in two previously reported cases [3, 4] and another one case of adenosarcoma with BAF features [5] indicate that BAF may originate from mesenchymal cells. Therefore, the clinical presentations, pathology and imaging manifestations of BAF remain to be explored.

We made a detailed analysis of clinical and pathological information of previous cases, and our current case was also included (Table 1). The cases consisted of 12 males and 15 females. Patients were aged from 23 to 83 years with median age of 57 years. The vast majority of symptoms were pain in the upper abdomen or asymptomatic. The physical examination and laboratory work-up of most patients were within the normal limits. Grossly, BAF is a well-circumscribed, nonencapsulated solid-cystic mass and has a white-purple surface. Histologically, BAF is comprised of tubular, microcystic and cystic structures lined by cuboidal to low columnar epithelial cells, embedded in a collagenous stroma. BAF harbors the potential for malignant transformation, but consistent criteria of its malignant histology have not been reported in the literature. We reviewed the pathologic data of all cases with malignant transformation [5–16], suggesting that malignant BAF may show the following characteristics: (1) columnar-type epithelial cells with disordered polarity; elongated, hyperchromatic, and vesicular nuclei with prominent nucleoli; eosinophilic cytoplasm with apocrine-like changes and secretory snouts; atypical mitotic figures. (2) complex papillary, cribriform-like, and back-to-back architecture. (3) stromal, perineural, lymphovascular, and liver capsule invasion. (4) cholangiocarcinoma arising in BAF. Immunohistochemically, the epithelial cells of BAF stained positive for CK7 and CK19, and the stroma cells stained positive for vimentin and SMA, negative for desmin. Ki67 proliferation index showed a significant difference between the benign and the malignant tumor components of BAF. In molecular pathology, the mutations of CDKN2A、CCND1、ERBB2、TP53 and KIT genes may contribute to tumorigenesis of BAF [4, 13, 16]. Most patients had a surgical resection. Except for 2 cases [4] that recurred for incomplete excision, all other patients with benign BAF had no recurrence or metastasis. Whereas, one case of a malignant BAF [6] had recurrence with abdominal wall invasion and multiple metastatic nodules in liver and lung at 3 years postresection. Therefore, more aggressive surgical procedures for the treatment of malignant BAF may improve the prognosis of patients compared with that of benign BAF.

**Table 1:** Literature review and analysis of pathological and clinical data

Author/Year	Sex	Age	Symptom	Physical examination/Laboratory work-up	Treatment	Immunohistochemistry/ Molecular studies	Follow-up	Malignant transformation
Tsui et al. [1]/1993	Female	74	RUQ pain	Liver function test normal	Wedge resection	Positive: cam5.2, AE1/3, EMA, CEA Negative: Chromo, S100, desmin, AFP, NSE	No recurrence after 20 years	No
Parada et al. [3]/1997	Female	49	RUQ pain	Liver function test normal	Partial hepatectomy	Monosomy 22	No recurrence	No
Akin et al. [6]/2002	Male	25	Abdominal enlargement, RUQ pain	RUQ palpable mass	Right lobectomy	–	Recurrence and pulmonary metastasis after 3 years	Yes
Garduño-López et al. [19]/2002	Female	68	RUQ pain, vomiting, diarrhea and jaundice	Liver enlargement Elevated CA19-9; hepatitis B surface Ag, ALP, total biliary, GGT, AFP normal	Left hepatectomy	Stained CA19-9	No recurrent after 30 months	No
Varnholt et al. [20]/2003	Female	47	RUQ pain, weight gain of 5 kg	–	Incomplete resection	Epithelial: positive for D10, p53 (50% to 75%), AE1/3, cam 5.2, CK7, CK19, CEA, EMA; negative for 1F6; Ki67 < 10% Stroma: positive for vimentin and SMA; negative for desmin	No metastasis or significant growth after 3 years	No
Gurrera et al. [31]/2010	Male	79	Vague abdominal pain	Blood tests and AFP normal	Partial resection	Epithelial: positive for CK7,CK8,CK9,CK19,EMA; negative for CEA,CK5/6,P53,calretinin, HBME-1, beta-catenin Stroma: positive for vimentin and SMA; negative for desmin	No recurrence after 7 years	No
Kai et al. [7]/2012	Male	40	Upper abdominal pain	Hematological, coagulation test,CEA, Ca19-9, AFP normal; carrier for HBV	Right hepatectomy	Positive: CK19, Ca19-9, MUC1 Negative: CEA, MUC2, MUC 5AC, p53, ER, PR, GCDFP15 Ki67 5% to 10%	Dying of fulminant hepatitis B 8 months after surgery	Yes
Nguyen et al. [8]/2012	Female	53	Incidentally found	Elevated CA-125; liver function tests, clotting profile, AFP, CA 19–9, and CEA normal	Segmental resection	Positive: CK7,CK19	No recurrent after 12 months	Yes
Tsutsui et al. [9]/2014	Female	69	Asymptomatic	General examination normal Complete blood count, chemistry, urinalysis, tumor markers, and coagulation normal	Partial liver resection	Epithelial: positive for CK7, CK19, CAM5.2, CKAE1/AE3, p53; negative for CEA, a-SMA; Ki67:10–15% in the dysplastic epithelia, 1–2% in non-dysplastic epithelia. Stroma: positive for vimentin and SMA; negative for desmin	No recurrent after 4 years	Yes
Jacobs et al .[10]/2015	Female	57	Incidentally found	Modest left costovertebral angle tenderness Mild leukocytosis, liver enzymes, CEA, AFP normal	Preoperative embolization and surgical resection	–	No recurrent after 5 years	Yes
Elpek et al. [32]/2016	Male	23	Asymptomatic	Physical examination normal Tumor markers, hematologic and coagulation normal	Partial hepatectomy	Positive: CK7, CK19, CK 18 and EMA Negative: AFP, PLAP, HCG, Hepatocyte, CK20, CD30, OCT4 and MUC2	–	No
Godambe et al. [11]/2016	Female	71	Bilateral upper abdominal pain	Liver function testing, serum alpha fetoprotein, CEA, and CA199 normal	Left hepatectomy	Positive: CK7 and CK19; brisk Ki67 P53(25% to 50%) Stroma: negative for Ki67 and p53 Negative: CD10, polyclonal CEA, Inhibin and PAX8	–	Yes
Thai et al. [12]/2016	Male	77	Fever, lumbosacral pain disorientation, and nocturnal agitation	Hyponatriemia, increase in inflammatory markers and anemia	Left lobectomy	Positive: CK7, CK19, CA19.9, CEA and MUC1	–	Yes
Thompson et al. [13]/2016	Male	71	Incidentally found	AFP, CA 19–9, Liver function tests, serology for HBV and HCV normal	Left hepatectomy	–	Dying for primary lung malignancy after 9 years	Yes
Thompson et al. [13]/2016	Male	71	Incidentally found	AFP, CA 19–9, Liver function tests, serology for HBV and HCV normal	Caudate lobe resection	Positive: CK7 Negative: CDX-2, CK20 CDKN2A mutation	No recurrent after 4 weeks	Yes
Kaminsky et al. [14]/2016	Female	37	Postprandial nausea, vomiting, and epigastric pain	–	Excising with wide local margins	Positive: CK7, CK19, synaptophysin, CD56 Negative: chromogranin, CK20, CDX2, heppar1, and p53 Ki67: 10% to 15% in BAF and 50% in CC	No recurrence after 4 months	Yes
Arnason et al. [4]/2017	4 females and 2 males	46 to 83	Abdominal pain (4 patients); incidental findings(2 patients)	–	Surgical resection(5 patients)	Positive: CKAE1/3, CK7, CK19,CA19-9 Ki67: less than 10% in the epithelial component, < 1% in the stromal component Amplifications of CCND1 and ERBB2	No recurrence in 3 patients after 3, 20, and 21 years Local hepatic recurrences in 2 patients after 1 and 6 years	No(series of 6 including 2 cases above)
Chua et al. [15]/2018	Female	66	Asymptomatic	AFP normal	Segmentectomy and adjuvant chemotherapy	Positive: CK7 Negative: CK20 and CDX2 Ki67: 2% BAF; CC 30%; P53 positive in BAF and CC	No recurrence after 6 weeks	Yes
Esteban et al. [33]/2018	Female	26	Jaundice and pruritus	Scleral icterus and generalized jaundice Elevated serum total bilirubin and alkaline phosphatase; AST, ALT, hepatitis serologies normal	Left hepatectomy	–	No recurrence after 3 months	No(co-existent BAF and hepatobiliary MCN)
Meguro et al.[5]/2018	Male	63	Found by MRI examination for liver cirrhosis	–	–	Epithelial: positive for CK7, CK19; negative for P53 Stroma: positive for vimentin, CD44, CD56,CD73,CD271;negative for P53, desmin, SMA Oseoblasts: positive for BMP-2	Dying for liver failure after 21 days	Yes(adenosarcoma)
Lee et al.[17]/2019	Male	63	Asymptomatic	Liver function tests,protein induced by vitamin K, AFP, antagonist-II, CA 19–9, and CEA normal	Bisegmentectomy	Epithelial: positive for CK7, CK19;P53(focally positive);Ki67(< 2%) Stroma: positive for SMA	No recurrence after 41 months	No
Lee et al.[17]/2019	Male	38	Asymptomatic	physical examination and tumor markers normal	Left lateral section	–	No recurrence after 39 months	No
Sturm et al. [16]/2019	Female	63	unspecific abdominal complaints	The physical examination and AFP, CEA, and CA 19–9 normal	Left hemihepatectomy	Epithelial: positive for CK7, Cadherin 17, CD56,Muc1 Stroma: positive for SMA Negative: inhibin, calretinin, S100P, ERG, Muc2, Muc4, Muc5, and Muc6 MLH1, MSH2, MSH6, and PMS2: nuclear expression Ki67: 5–10% in biliary adenofibroma; 20–30% in the adenocarcinoma Different polymorphisms in the encoded TP53 and KIT	No recurrence after 24 months	Yes
Present case	Female	64	RUQ pain	Complete blood count, chemistry, coagulation, liver function test, tumor markers(AFP、CA125、CA19-9、CA15-3 and CA72-4)normal Hepatitis B surface antigen, e antibody, core antibody positive	partial resection	Positive: CK7、CK19、CEA KI67: less than 10% in benign part; 20–30% in malignant part	No recurrence after 9 months	Yes

*RUQ* right upper quadrant

Symptoms and laboratory data of BAF are nonspecific, making it difficult to differentiate BAF from other more common hepatic lesions. While pathologic diagnosis by liver biopsy is regarded as the gold standard for diagnosis, it is not without limitations. Liver biopsy is an invasive procedure with the risk of various complications, such as bleeding, seeding the tract, infection, etc. Sampling error is also an issue with liver biopsy, especially for cystic lesions which are prone to a false-negative diagnosis. These limitations emphasize the importance of developing sensitive and specific imaging techniques to diagnose BAF. However, the vast majority of case reports in the literature only focused on the clinical and pathological features of BAF, and lacked detailed professional descriptions of the imaging manifestations. Thus, we reviewed 15 reports with detailed imaging information and summarized the imaging features on US, CT and MRI of 17 patients with BAF (Table 2). To our knowledge, this is the first detailed comprehensive review of the imaging characteristics of BAF in the published literature. Lee et al. [17] simply summarized the MRI findings of 8 patients with BAF, but not the radiologic features of other imaging modalities or the enhancement patterns of lesions.

**Table 2:** Literature review and radiological data analysis

Author/Year	Malignant transformation	Location	Number of lesions	Size(cm)	Shape	Margin	US	CT	MRI	Internal septa	Enhancement	Liver contour	Bile duct communication
Akin et al. [6]/2002	Yes	Subcapsular area of right lobe	Multiple	14	Lobulated	Obscure	—	—	—	Unilocular	Enhancement in arterial phase and early washout in portal phase	Protrusion	No
Garduño-López et al. [19]/2002	No	Subcapsular area of left lobe	Solitary	6	Lobulated	Well-defined	hypoechoic lesion	Solid-cystic mass	—	Multilocular	—	Protrusion	No
Varnholt et al. [20]/2003	No	Subcapsular area of left lobe	Solitary	16	Lobulated	—	cystic and solid mass with areas of increased echogenicity	Solid-cystic mass	—	Multilocular	—	Protrusion	No
Kai et al. [7]/2010	Yes	Subcapsular area of right lobe	Solitary	7	Lobulated	Well-defined	—	Multicystic mass lesion	—	Multilocular	Gradual enhancement	Protrusion	No
Tsutsui et al. [9]/2014	Yes	Subcapsular area of right lobe	Solitary	3.5	Lobulated	—	hyperechoic nodule with small hypoechoic	hypodense solid-cystic mass;	T1WI: low intensity; T2WI: high intensity and low intensity septa; DWI: markedly high intensity	Multilocular	One part: enhancement in arterial phase and early washout in portal phase; another part: early and prolonged enhancement	Protrusion	No
Jacobs et al. [10]/2015	Yes	Subcapsular area of right lobe	2	11.8	Lobulated	—	—	Heterogeneous, predominantly hypodense mass	—	Multilocular	—	Protrusion	No
Elpek et al. [32]/2016	No	—	Solitary	6	—	Well-defined	—	Multicystic mass lesion containing solid areas	—	Multilocular	—	—	—
Godambe et al. [11]/2016	Yes	Subcapsular area of left lobe	Solitary	6.3	—	—	—	—	—	Multilocular	Heterogeneous enhancement in arterial phase	—	—
Thai et al. [12]/2016	Yes	Subcapsular area of left lobe	Solitary	4	Targetoid	—	—	A targetoid lesion with a peripheral edematous halo and a necrotic central area	—	Unilocular	—	—	No
Thompson et al .[13]/2016	Yes	Subcapsular area of left lobe	Solitary	14.5	Lobulated	Well-defined	—	—	T2WI: heterogeneously increased signal; T1WI:isointensity to hypointensity	Multilocular	Enhancement in arterial phase and early washout in portal phase some regions: retention of contrast in delayed phase	Protrusion	No
Thompson et al.[13]/2016	Yes	Subcapsular area of caudate lobe	Solitary	6.6	Lobulated	Well-defined	—	—	T2WI: heterogeneously increased signal; DWI: restricted diffusion	Multilocular	Peripheral enhancement on delayed imaging	Protrusion	No
Kaminsky et al. [14]/2016	Yes	Subcapsular area of right lobe	Solitary	4.9	Lobulated	—	—	—	T1WI: hypointense, T2WI: heterogeneously hyperintense	Multilocular	Peripheral enhancement	Protrusion	No
Chua et al. [15]/2018	Yes	Subcapsular area of left lobe	Solitary	—	—	—	—	—	DWI: restricted diffusion	—	Enhancement in arterial phase and early washout and pseudocapsule formation in portal phase	—	—
Lee et al. [17]/2019	No	Subcapsular area of segments IV and VIII	Solitary	4.7	Lobulated	Well-defined	—	—	T1WI: low signal intensity; T2WI: bright signal intensity tumor with hypointense septa	Multilocular	Septal enhancement in delayed phase	Protrusion	No
Lee et al. [17]/2019	No	Subcapsular area of left lobe	Solitary	2.7	Lobulated	Well-defined	—	low attenuation	T1WI: hypointensity; T2WI: bright signal intensity	Multilocular	Septal and wall enhancement in portal venous phase	Protrusion	No
Sturm et al. [16]/2019	Yes	Subcapsular area of left lobe	Solitary	6.3	Lobulated	—	—	Solid-cystic mass	—	Multilocular	—	Protrusion	No
Present case /2021	Yes	Subcapsular area of right lobe	Solitary	1.8 cm	Irregular	Well-defined	hypoechoic subcapsular nodule	Hypodense solid-cystic mass	—	Unilocular	Enhancement in arterial phase and early washout in portal phase	Retraction	No

Because of wide availability, low cost and nonradiative, conventional US is the screening method of the choice. The sensitivity for US in the diagnosis of liver cystic lesions is in the range of 90% [18]. However, US characteristics of BAF were not mentioned in most literature. Only 2 patients [19] showed hypoechoic masses, and another 2 patients [9, 20] were presented as hyperechoic masses. These US manifestations are nonspecific, and two cases were misdiagnosed as hemangiomas. US can diagnose common liver lesions with confidence, but its’ ability in the evaluation of complex cystic lesions, such as rare BAF, is limited. Due to the lack of enhancement patterns, many different types of liver lesions can't be differentiated in US. Contrast-enhanced ultrasound (CEUS) is an emerging technique in liver imaging. By using a microbubble agent as contrast, this modality can provide detailed information about tumor architectures and allow observations of enhancement patterns in real-time. The high diagnostic accuracy of CEUS for focal liver lesions has been reported in several studies [21], which may be helpful in characterizing BAF.

CT has become the most commonly used modality in the preoperative assessment and follow-up of the patients with hepatic tumors. For liver cystic lesions, CT can better demonstrate gas contents and calcification within the cyst. On CT, BAF may appear as a solitary, large hypodense solid-cystic mass with a well-defined margin, lobulated shape and internal septa. The tumor abuts the liver capsule and has a protruding liver contour. No communication is observed between the tumor and intrahepatic bile ducts. Notably, our current case is the first report of BAF with capsular retraction, which may be affected by the distribution of fiber components. And our patient showed multiple hypoattenuating lesions with sizes < 0.5 cm, irregular outlines and obscure margins in the subcapsular area of the liver, which are similar to von Meyenburg complexes [22]. In staining pattern and histology, there is a striking resemblance between BAF and biliary hamartomas. Varnholt et al. [20] suggested that BAF possibly represents transformed von Meyenburg complexes. And 2 case reports [5, 11] of BAF showed von Meyenberg complexes existed in the postoperative specimens of background liver but didn’t record their imaging findings. Thus, we considered that von Meyenberg complexes in the background liver may be a typical but rare imaging feature for BAF diagnosis.

MRI has been considered as the most useful modality for characterizing liver masses, due to its high soft-tissue contrast resolution. In MR imaging, BAF appears as a solitary, subcapsular, multiseptated solid-cystic mass with low signal intensity on T1-weighted images and high signal intensity on T2-weighted images. Other imaging features of BAF in MRI, such as large size, well-defined margin, lobulated shape and no intrahepatic bile duct communication, are similar to those on CT. The principal advantage of MRI over CT for liver cystic lesions is its better visualization of the mural nodule, hemorrhage and mucin within the cyst. However, histological studies showed that the cysts of BAF were non-mucinous type and intratumoral hemorrhage was uncommon [9]. And mural nodules within the tumor have never been reported. Therefore, MRI can be helpful for characterizing BAF, but it does not provide additional information compared with CT.

Benign BAF can be curative after complete surgical resection, while malignant BAF has a risk of local recurrence and distant metastasis. Therefore, the aggressiveness assessments of BAF before surgery are of great importance. On unenhanced images, the imaging findings of most BAF with malignant features resembled those of benign BAF. Yet, it was found that several features showed in some malignant BAF cases, including multiple lesions, unilocular solid-cystic mass, restricted diffusion on DWI, obscure margin, peripheral edematous halo and pseudocapsule formation, have not been described in benign BAF [6, 10, 12, 15]. As for the enhanced characteristics of BAF, 10 cases offered detailed information of imaging features on contrast-enhanced CT or MR images, including 2 cases of benign BAF and 8 cases of malignant BAF. 2 patients [17] with benign BAF (100%) showed delayed enhancements, and 6 patients [6, 9, 11, 13, 15] with malignant BAF (75%) showed marked enhancements in the arterial phase and washout in the venous phase. Thus, we hypothesize that wash-in the arterial phase followed by wash-out in the venous phase is a typical imaging feature of malignant BAF. Delayed enhancement of benign BAF may be related to the high content of fibrous stroma. Malignant BAF has a complex architecture with crowded, back-to-back tubular structures, and lacks the fibrous stroma, which may be the reason leading to early enhancement. In addition, 2 of the 6 cases of malignant BAF mentioned above [9, 13] showed prolonged enhancements in some regions of the tumor, and two reports [7, 13] (25%) described malignant BAF with delayed enhancements. That may be associated with the varying degrees and ranges of malignant transformation. The various key imaging findings which may help in distinguishing benign and malignant BAF, were summarized in Table 3. Because BAF is extremely rare, the further investigations of its enhancement characteristics are required. Dual-energy computed tomography (DECT) is a promising approach in the evaluation of liver lesions. Based on CT data at two different energy spectra, DECT can yield several types of images including virtual monoenergetic imaging, effective atomic number map, iodine map and so on, which is particularly useful to improve iodine contrast visualization and quantitatively reflect the blood flow [23]. DECT increases the accuracy in the differentiation between benign and malignant hepatic lesions through iodine quantification [24]. In addition, MRI can evaluate focal liver lesions in both the dynamic and hepatocyte phases by using hepatocyte-specific contrast agents (HSCAs) [25]. Malignancy should be considered when hypervascular lesions appear hypointense in the hepatocyte phase [26]. These emerging approaches in liver imaging can provide more information about enhancement characteristics of focal liver lesions, and might be helpful in differentiating benign and malignant BAF.

**Table 3:** Different imaging features of benign and malignant BAF

Imaging feature	Benign BAF	Malignant BAF
Number of lesions	Solitary	Multiple
DWI signal	—	Restricted diffusion
Margin	Well-defined	Obscure
Internal septa	Multilocular	Unilocular
Enhancement	Delayed enhancement	Marked enhancement in the arterial phase followed by wash-out in the venous phase
Additional features	—	Peripheral edematous halo, pseudocapsule formation

The distinct imaging features can differentiate BAF from other liver cystic lesions: (1) Liver abscess [27]: abscesses usually appear as thick-walled cystic lesions with perilesion edema. The presence of internal gas is a typical imaging characteristic of the abscess. After contrast injection, the rim enhancement of lesion and hypodense perilesion edema form the so-called “ring sign”. And the patients with liver abscess often present infection symptoms such as high fever, shiver and leukocytosis. (2) Hepatic cyst [28]: hepatic cysts appear as round cystic lesions with thin walls, smooth outlines and no internal septa. No enhancement is seen after the administration of contrast material. (3) Cystic metastases [29]: metastatic tumors with obvious necrosis and cystic degeneration are regarded as cystic metastases. Cystic metastases usually appear as multiple, round, unilocular cystic lesions. Enhancing mural nodules and peripheral rim can be observed in contrast-enhanced images. In addition, the medical history of primary malignancy can help reach a correct diagnosis. (4) Cystic Hepatocellular Carcinoma [22]: cystic hepatocellular carcinoma usually occurs in the context of cirrhotic liver. The wall of cyst caused by internal necrosis has an irregular thickness. Elevated AFP also can suggest the diagnosis. (5) Intraductal papillary neoplasm of the bile duct (IPNB) [29]: IPNBs appear as soft tissue masses within the dilated bile ducts. The morphology of intraluminal mass and the degree of dilated bile ducts are various. MR cholangiography depicts the relationship of the lesion to the bile ducts well and therefore contributes to the diagnosis. (6) Mucinous cystic neoplasm (MCN) [30]: MCNs appear as uni- or multilocular cystic tumors with irregular thick walls and internal septations. Mural nodules, hemorrhage or calcification within the cyst can be observed. MCN can present hyperintense on T1 weighted images due to its mucin production. The lesions demonstrate no or mural nodular enhancement on postcontrast enhanced images.

In conclusion, BAF is a rare hepatic tumor with the potential of malignant transformation, which requires prompt treatments and follow-ups. Although symptoms and laboratory data of BAF are nonspecific, CT and MRI may help in diagnosing BAF and evaluating its aggressiveness before surgery. The current case and literature review suggest that BAF is radiologically characterized by the following features: (1) abutting the liver capsule; (2) solitary, large solid-cystic mass with a well-defined margin, lobulated shape, internal septa; (3) no communication between the lesion and intrahepatic bile ducts; (4) von Meyenberg complexes in background liver may be a typical but rare imaging feature; (5) enhancement patterns may have the potential to assess the aggressiveness of BAF and that marked enhancement in the arterial phase followed by wash-out in the venous phase is suggestive of malignant BAF. In addition, further investigations on the role of emerging approaches, including CEUS, DECT and MRI with HSCAs, in characterizing BAF are required.

## Data Availability

All data generated or analyzed during this study are included in this published article.

## Abbreviations

AFP: Alpha-fetoprotein
CA: Carbohydrate-antigen
MRI: Magnetic resonance imaging
CT: Computed tomography
CK: Cytokeratin
CEA: Carcinoembryonic antigen
WHO: World Health Organization
SMA: Smooth muscle actin
CDKN2A: Cyclin-dependent kinase inhibitor
CCND1: CyclinD1
ERBB2: Her2/neu gene
